# The Great Belt train accident: the emergency medical services response

**DOI:** 10.1186/s13049-021-00954-7

**Published:** 2021-09-23

**Authors:** Peter Martin Hansen, Søren Bruun Jepsen, Søren Mikkelsen, Marius Rehn

**Affiliations:** 1 The Mobile Emergency Care Unit, Department of Anaesthesiology and Intensive Care Medicine, Odense University Hospital Svendborg, Baagøes Allé 31, 5700 Svendborg, Denmark; 2 Danish Air Ambulance, Olof Palmes Allé 34, 1. Sal, 8200 Aarhus N, Denmark; 3 The Mobile Emergency Care Unit, Department of Anaesthesiology and Intensive Care, Odense University Hospital, J. B. Winsløws Vej 4, 5000 Odense, Denmark; 4 The Prehospital Research Unit, Region of Southern Denmark, Odense University Hospital, J. B. Winsløws Vej 4, 5000 Odense, Denmark; 5 Department of Regional Health Research, University of Southern Denmark, Campusvej 55, 5230 Odense, Denmark; 6 Department of Research and Development, Norwegian Air Ambulance Foundation, Postboks 414 Sentrum, Oslo, Norway; 7 Air Ambulance Department, Division of Prehospital Services, Oslo University Hospital, Kirkeveien 166, 0450 Oslo, Norway; 8 Faculty of Health Sciences, University of Stavanger, Kjell Arholms Gate 41, 4021 Stavanger, Norway

**Keywords:** Major incident management, Mass casualty incidents, Communication

## Abstract

**Background:**

Major incidents (MI) are rare occurrences in Scandinavia. Literature depicting Scandinavian MI management is scarce and case reports and research is called for. In 2019, a trailer falling off a freight train struck a passing high-speed train on the Great Belt Bridge in Denmark, killing eight people instantly and injuring fifteen people. We aim to describe the emergency medical services (EMS) response to this MI and evaluate adherence to guidelines to identify areas of improvement for future MI management.

**Case presentation:**

Nineteen EMS units were dispatched to the incident site. Ambulances transported fifteen patients to a trauma centre after evacuation. Deceased patients were pronounced life-extinct on-scene. Radio communication was partly compromised, since 38.9% of the radio shifts were not according to the planned radio grid and presented a potential threat to patient outcome and personnel safety. Access to the incident site was challenging and delayed due to traffic congestion and safety issues.

**Conclusion:**

Despite harsh weather conditions and complex logistics, the availability of EMS units was sufficient and patient treatment and evacuation was uncomplicated. Triage was relevant, but at the physicians’ discretion. Important findings were communication challenges and the consequences of difficult access to the incident site. There is a need for an expansion of capacity in formal education in MI management in Denmark.

**Supplementary Information:**

The online version contains supplementary material available at 10.1186/s13049-021-00954-7.

## Background

Major Incidents (MI) defined as incidents that require the mobilization of extraordinary emergency medical services (EMS) resources [1] are rare occurrences in Scandinavia and the literature on disaster epidemiology is scarce [2]. When MI do occur, they are subjected to massive media coverage and public interest [3, 4]. Incidents such as the 22nd of July 2011 Oslo/Utøya disaster in Norway [5, 6], school shootings and lone terrorist attacks in Finland, Sweden, and Denmark, and the shipwrecks of Scandinavian Star [7] and Estonia [8], highlight the relevance of MI preparedness in the EMS.

On January 2nd, 2019, a high-speed passenger train collided with a trailer falling from a freight train on the Great Belt Bridge near Nyborg, Denmark. Eight people were killed instantly, and fifteen patients were injured and brought to a trauma center.

The Great Belt train accident prompted a massive EMS response and involved responders from multiple authorities, private and state contractors. The incident turned out to be the most severe MI in Denmark for thirty years. We aim to describe the immediate prehospital EMS response to the Great Belt Train Accident and evaluate adherence to guidelines to identify areas of improvement for future MI management.

## Material and methods

### Danish EMS

Denmark is a Scandinavian country consisting of the Jutland peninsula, as well as 406 islands of varying sizes; the main islands Funen and Zealand interconnected with bridges and connected with Sweden as well. Denmark has a temperate climate with relatively cool summers and moderately cold winters. The area covers 43.094 square kilometers with a population of approximately 5.8 million inhabitants [9]. Denmark is divided into five health regions with council members elected by the people. The regions are responsible for the Danish Health Care System [10]. The Danish health care system includes the prehospital service and is publicly funded, and thus free of charge at the point of care.

Each region has its own EMS agency including an Emergency Medical Communication Centre (EMCC). The regional EMS is responsible for dispatch, treatment, triage, and transport of all patients from the emergency call made to national emergency number 1-1-2 is received at the EMCC until the patient has been handed over to the hospital staff or treatment has been completed on-scene. EMS response relies on a systematic criteria-based dispatch protocol [11]. This protocol has been described in detail elsewhere [12].

The EMS in Denmark is a three-tiered system consisting of ambulances manned by a combination of emergency medical technicians (EMT) with basic, intermediate, or paramedic level of training. The Danish EMS also includes rapid response units manned by paramedics and mobile emergency care units (MECU) staffed by specialists in anaesthesiology with sub-specialization in prehospital critical care. A supplementary physician-manned helicopter EMS (HEMS) is also available. Approximately 300 ambulances, 26 MECUs, and four HEMS helicopters are available in the Danish EMS (Table 1) [13, 14].

**Table 1:** Overview of Danish MECU/HEMS units and Consultants’ Major Incident Course as of 01.06.21

	MECU and HEMS units	Total Consultants	Major Incident Course	Percentage
Capital	5	76	43	56.58
Zealand	2	27	21	77.78
South Denmark	6	90	40	44.44
Central Denmark	10	148	51	34.46
North Denmark	3	42	22	52.38
Danish Air Ambulance	4	46	46	100.00
Total	30	429	223	51.98

MECU, Mobile Emergency Care Unit; HEMS, Helicopter Emergency Medical Service

In addition, the Royal Danish Air Force Squadron 722 utilizes three Search and Rescue (SAR) EH-101 Merlin helicopters on 24/7/365 duty, managed by the Joint Rescue Coordination Centre in Aarhus that has a role in MI or incidents at sea or near seawater.

### The Danish Trauma system

Denmark has a two-tiered trauma system comprising both regional hospitals and university hospitals. The catchment areas differ between the five regions. The four university hospitals in Copenhagen, Odense, Aarhus, and Aalborg are all trauma referral centers, each providing definitive care for a population of 500.000 to 2.800.000 people.

### Major incident preparedness

The Danish guidelines for interdisciplinary MI management [Retningslinjer for Indsatsledelse, REFIL] [15] is a theoretical and practical framework for a national, interdisciplinary MI management concept. The theoretical mutual education in MI management for Police, Fire & Rescue, and Medical Incident Commanders comprises a three-week course at the Danish Emergency Management Agency (DEMA) training facility. The course encompasses all aspects of MI management and includes numerous tabletop exercises and a full-scale exercise. After passing a comprehensive exam, the course is completed.

The national crisis management system serves to provide government coordination of the response to incidents in Denmark or abroad. Prevention includes an overview, risk assessment, management planning, risk communication, and awareness. Preparedness relies on training, exercises, and early warning systems, currently restricted to weather-related issues.

Emergency management and response rely on sector responsibility, meaning that the authority or organization with the daily responsibility for a certain area also carries this responsibility during emergencies. The Danish Health Authority (DHA) is the overall national authority for emergency preparedness in the health sector.

On the strategic level, the National Operational Staff, which includes the Danish National Police (chair), DEMA, DHA, The National Defence Command, the Police Intelligence Services, and further resources depending on the task, is established in the event of a national or international crisis.

On the operational level, twelve Local Operational Staffs with permanent members from the involved police district (chair), DEMA, the Home Guard, the health region in question, and the municipalities, are available for activation in the event of a local emergency. Ad hoc participants are often included depending on the scenario.

On the tactical level, a Joint Incident Command comprising Police, Fire & Rescue, and Health is responsible for the overall incident command. An ambulance scene commander is responsible for the coordination of the EMS units. The Casualty Clearing Station Officer performs secondary triage, coordinates treatment and allocation of patients with the EMCC physician, and refers to the Medical Incident Commander in the Joint Incident Command.

In a MI, an on-call physician joins the EMCC; coordinates the allocation of patients to hospitals with the on-scene Medical Incident Commander, and coordinates the allocation of supplementary resources, e.g. cleansing stations, additional ambulances, or physicians. The receiving hospitals are obliged to have contingency plans for mass casualty handling when a MI is declared.

**Fig. 1:**
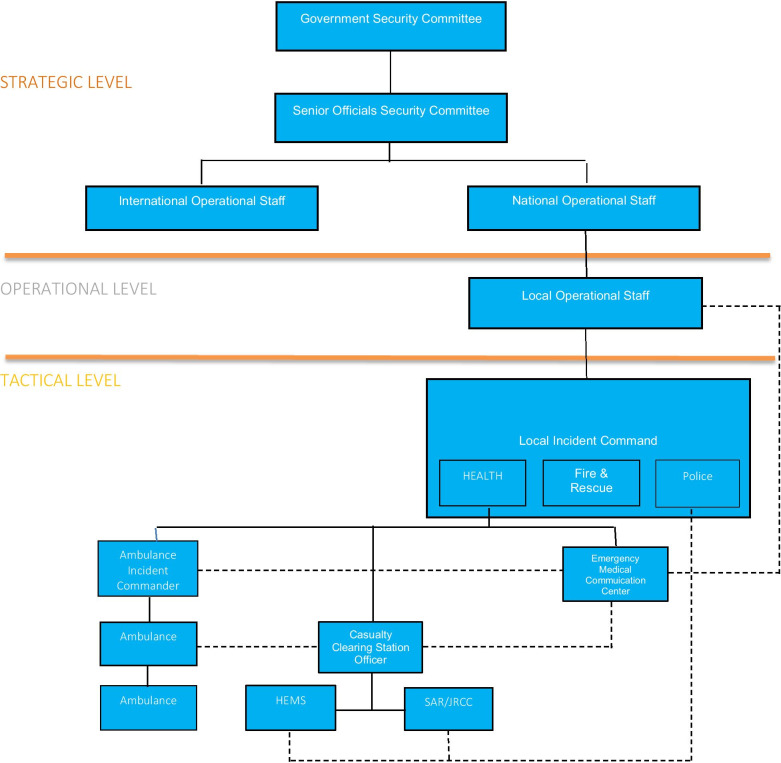
Danish National Crisis and Major Incident Management System. HEMS, Helicopter Emergency Medical Service; SAR, Search & Rescue; JRCC, Joint Rescue Coordination Center

Figure 1 depicts the Danish National Crisis and Major Incident Management System.

### Communication

Danish emergency services dealing with public order, safety, and health use the nationwide emergency radio network, called SINE (Safety Network), a digital radio network based on the Terrestrial Trunked Radio [16] TETRA standard. The responsibility to secure the operation and development of the SINE is in the hands of the Centre of Emergency Communication [17] (CFB), a part of the Danish National Police.

In case of an incident involving multidisciplinary units, the Danish police issues a temporary interdisciplinary communication channel group within the SINE system. Large structures like the airports in Copenhagen, Aalborg and Billund, the Danish Parliament, and the Øresund and the Great Belt Connections have predefined interdisciplinary communication channel groups.

### Patient Management and documentation

The prehospital electronic patient record system in Denmark features an electronic casualty clearing station record that is available for EMS units involved in a MI. The prehospital electronic patient record system serves as the unique identification tool. Unique identification is ensured when a wristband placed on MI patients is scanned into the portable tablet at the scene. This record enables the registration of the injuries and the triage levels of the patients at the scene of the incident and thus provides an overview of the patients arriving from a MI to a trauma center (Additional file 2).

### Scene description

**Fig. 2:**
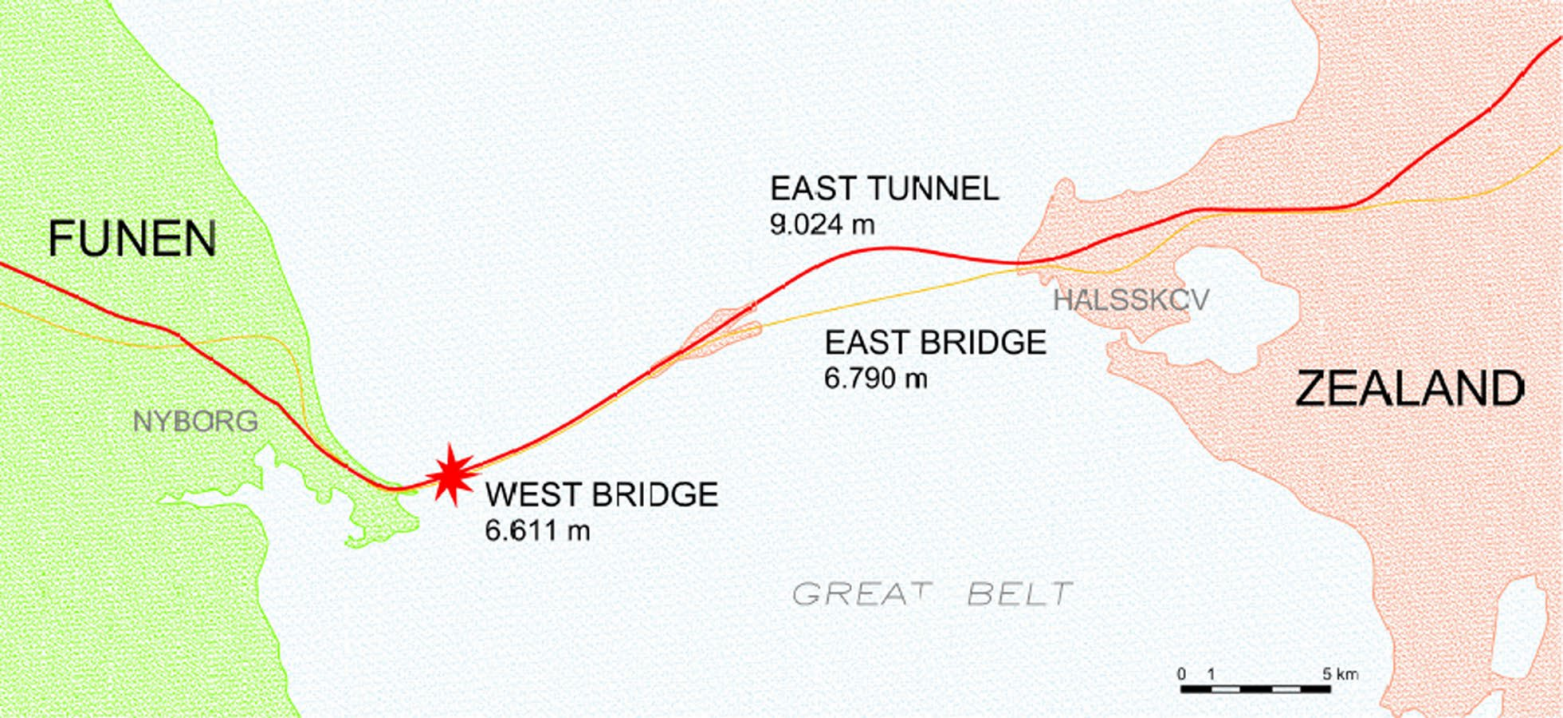
Map of incident scene

**Fig. 3:**
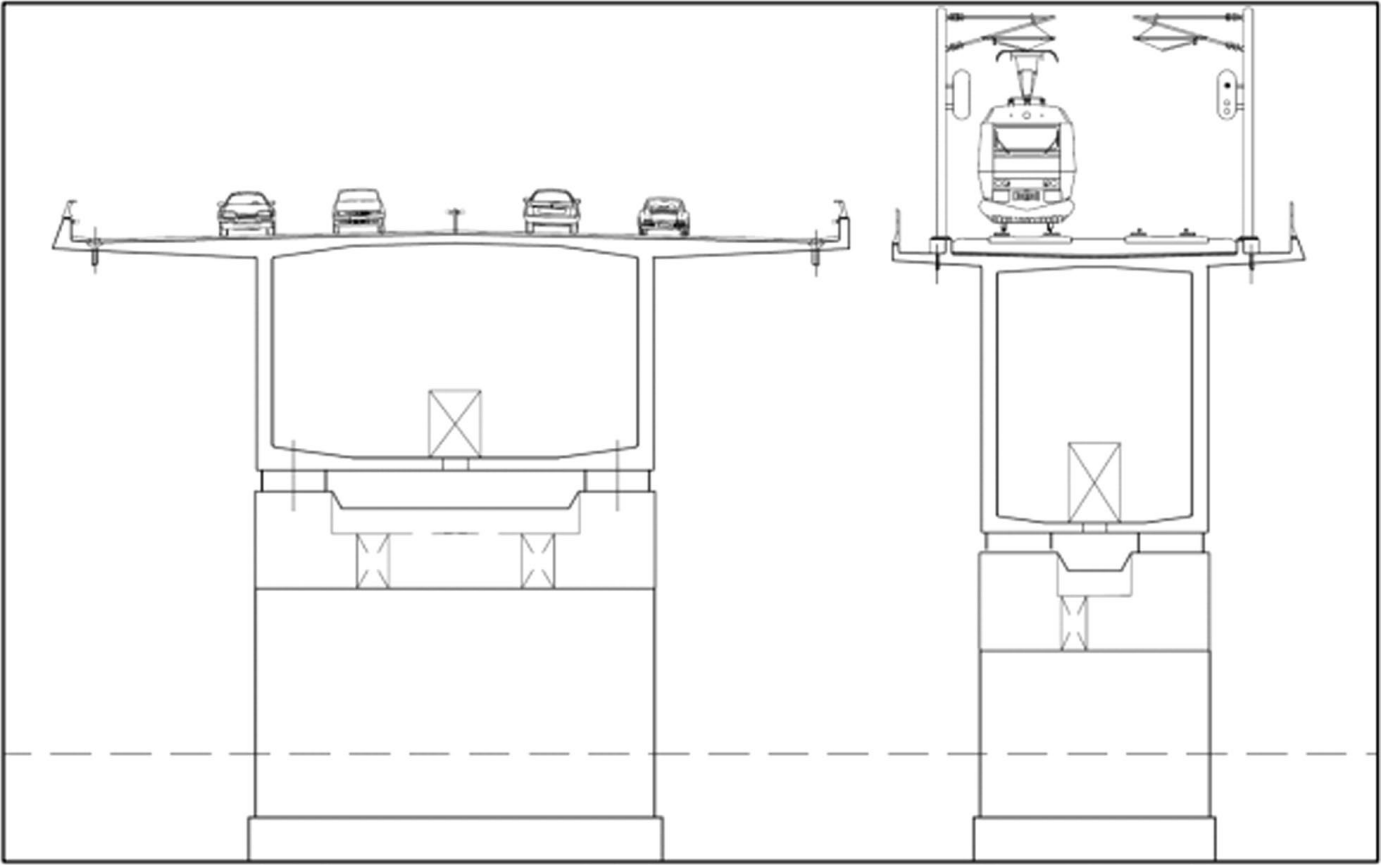
Cross-section of bridges. Distance between bridges is 1.8 m

The eighteen-kilometers-long fixed link across the Great Belt comprises two bridges and a tunnel (Fig. 2). The East Bridge between Zealand and Sprogø is 6790 m long and spans the Great Belt Eastern Channel, an international waterway. The passage height is 65 m. Sprogø in the middle of the Great Belt connects the bridges and the tunnel. The East Tunnel for rail traffic is 8024 m long and comprises two separate tunnel tubes each containing one track. The West Bridge, which is a combined road and rail bridge, is 6611 m long and consists of two parallel bridges, one for road and one for railway traffic. Passage height is 18 m above sea level. The road bridge and the rail bridge are situated 1.8 m apart with fixed passages between road and railways for every 550 m (Fig. 3).

The high-speed train L210 was of the IC4 type, a diesel-electric four-coach composing of two train sets. The passenger capacity was 204 per train set. On the day of the incident, the L210 carried 131 passengers and three crew. The Danish State Railways operated the train that was en-route from Aarhus to Copenhagen Airport Kastrup.

Deutsche Bahn Cargo Scandinavia® operated the cargo train G9233. It comprised one electric locomotive pulling eight six-axle pocket wagons loaded with ten trailers in total. It had left Høje Taastrup for Fredericia at 06.30.

The distance from the incident site to Odense University Hospital is 41 km; the ground transport time is approximately thirty minutes. Odense University Hospital is a trauma referral center for the 1.223.000 inhabitants of the Region of Southern Denmark. The region’s ambulance operator Ambulance Syd operates fourteen ambulance stations with twenty-three ambulances on the island of Funen. The neighboring region on Zealand operates approximately fifty-five ambulances from twenty-six stations. MECUs are located in Odense, Svendborg and Slagelse. The HEMS bases are located in Ringsted on Zealand and in Billund, Skive and Aalborg in Jutland. The EMCCs located in Odense and Slagelse coordinate the EMS units. SAR bases are in Aalborg, Skrydstrup and Roskilde.

### Study design

This is a retrospective and observational study of the EMS activity on January 2^nd^, 2019 following the Great Belt train accident. The epidemiological assessment of this MI adheres to the CONFIDE (CONsensus guidelines on Reports of Field Observations in Disasters and Emergencies) [18] guidelines. CONFIDE constitutes a method of assessing the quality of non-traditional studies for the acquisition of the best possible evidence approach to response in a disaster.

The medical directors of the involved EMS, Danish Air Ambulance; heads of research of Odense University Hospital Odense and Svendborg, and Chief executive officer of the Region of Southern Denmark approved the research project and allowed data collection from EMCC logs and CFB. The research directors approved the study as a quality improvement project; therefore, formal approval from the Regional Committee for Medical and Health Research Ethics was unnecessary after consultation (S-20212000-43 Acadre 21/209).

### Data acquisition and variables

Data sources, including information on EMS activity, communication, and patient management were:

- Control Room System (SimaTech®, Ballerup, Denmark), EMCC, Region of South Denmark, Odense, DK
- Control Room System (Carmenta®, Gothenburg, Sweden), EMCC, Region Zealand, Slagelse, DK
- Prehospital Patient Journal (Judex®, Aalborg, Denmark), Regions of Denmark, Copenhagen, DK
- Centre of Emergency Communication, Frederiksberg, Copenhagen, DK
- HEMS File, Danish Air Ambulance, Prehospital Services, Aarhus, DK
- Royal Danish Air Force Joint Rescue Coordination Centre, Aarhus, DK
- Danish Meteorological Institute, Copenhagen, DK
- Infomedia, Copenhagen, DK
- Public domain

## Results

### Meteorological conditions

At the time of the MI at 07:29, the mean wind speed at the incident site was 14.8 m/s with gusts at 20.5 m/s. The wind direction was between 340 and 350 degrees, thus perpendicular to the West Bridge. The temperature was 4.4 degrees Celsius. The wind chill temperature was between -3 and -4 degrees Celsius in gusts. It was sky clear; twilight was to be at 07:57 and sunrise at 08:40.

### Flooding conditions

The Danish Meteorological Institute had issued a flood warning for the southern waters of Denmark. Following several days with the wind coming in from the North, water had collected in the Baltic Sea, and therefore, a flood warning of 140 to 165 cm flood above daily sea level had been issued. This prompted the assembly of the Local Operational Staff in the Funen Police district at 07:00 on the morning of the incident.

### Alarm and dispatch

**Fig. 4:**
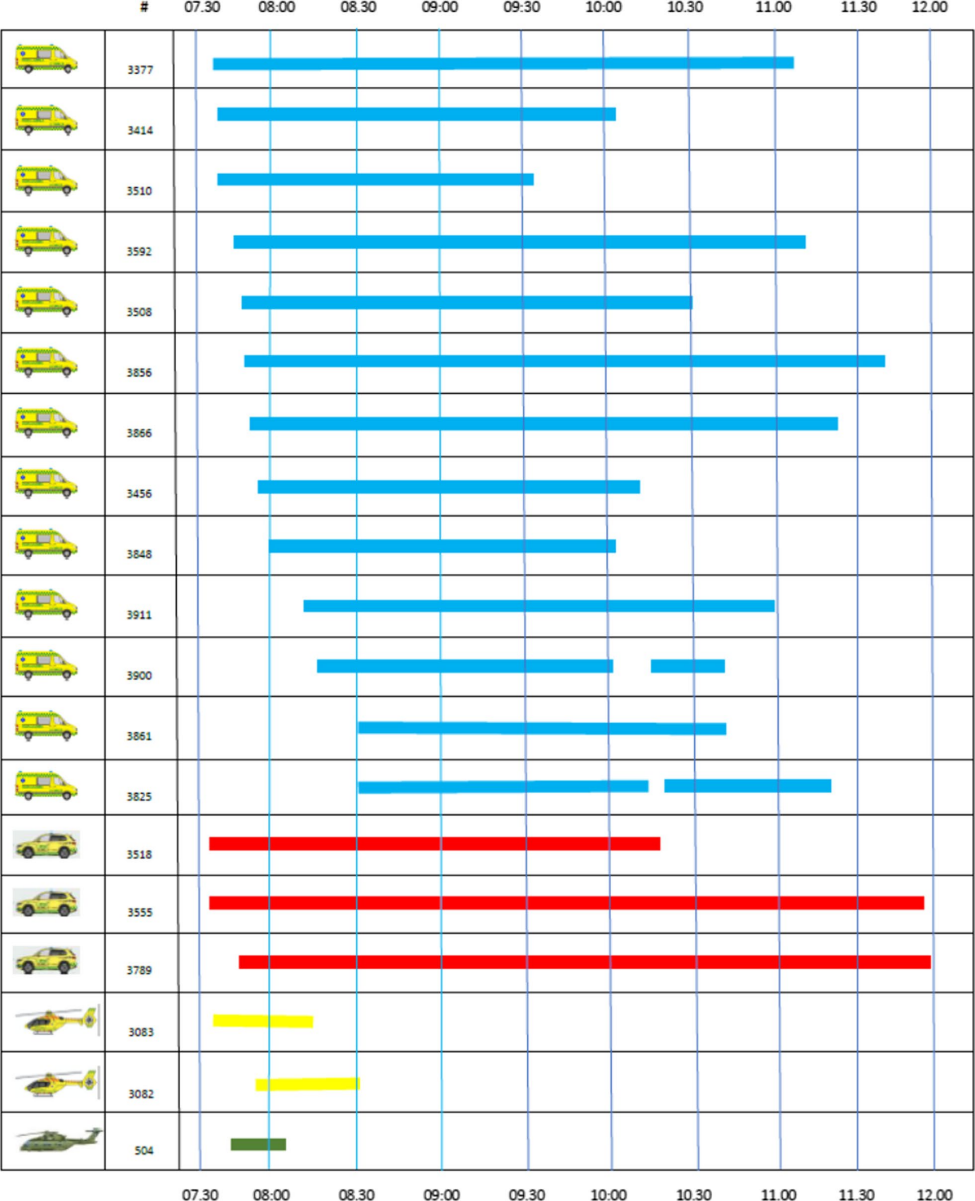
Overview of EMS units and time expenditure

At 07:29, a loose trailer hanging from the freight train G9233 struck the high-speed train L210. The Danish national emergency number 1-1-2 received the first call from a passenger on the train at 07.33. The health care officer taking the call acknowledged that a MI had taken place and scrambled the first units at 07:37. In sequence, thirteen ambulances, three MECUs, two HEMS helicopters, and one SAR helicopter from the Joint Rescue Coordination Centre were dispatched to the scene. Figure 4 displays the sequence and time expenditure of the units (Additional file 1).

### Site access

Due to the weather conditions, The Great Belt Bridge was closed for road traffic at 03.18. Consequently, massive congestion was present at both ends of the bridge, making access to the Great Belt Bridge a challenge. Police directed the first units to use the closed-down westbound motorway to drive east to the West Bridge. The congestion delayed several units in their arrival at the incident scene.

The railway bridge is separated from the motorway bridge by a gap of 1.8 m with connecting passageways for every 550 m. The L210 was situated between two of these passageways. Power lines carrying up to 25kVolts had been severed and were lying on the ground. This represented a potential danger to the rescue personnel who could not access the train bridge until the power was disconnected centrally and the power lines manually grounded by the Fire & Rescue services. Two pre-made aluminum bridges for evacuation of passengers stranded between the permanent passageways had to be picked up from storage facilities by a truck with a crane. The heavy wind complicated the handling of the light bridges. To secure the portable aluminum bridges the railings of the neighboring bridges had to be cut, leaving free access to the sea below.

### Site organization

**Fig. 5:**
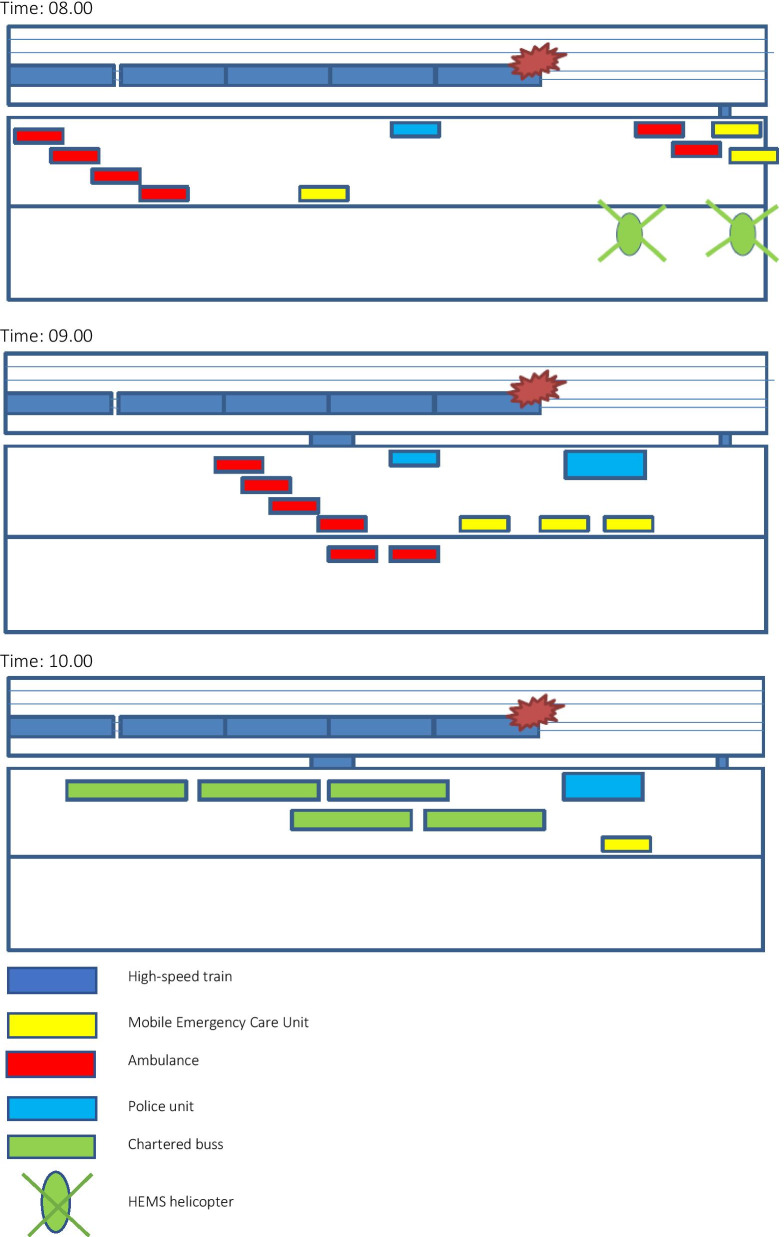
Organization of incident site in sequential time

The closed westbound motorway track opposite the train was in use for the line-up of EMS, Police, and Fire & Rescue units, alleviating the need to shut down traffic and the concern for scene safety. The casualty clearing station was defined as the ambulance vehicles, obviating the need for a physical space designated as a casualty clearing station. Thus, the initial treatment took place inside the ambulances and during transfer to the hospital. The inner cordon (the defined workspace for the fire and rescue department) was set around the L210 train. The two HEMS helicopters landed on the eastbound motorway track 50–100 m from the train. The incident site organization is displayed in Fig. 5 (Additional files 3, 4).

### Triage

When the scene was declared safe by Police and Fire & Rescue, two rescue teams consisting of one EMS physician and two EMT/Paramedics entered the train from the east end and triaged the patients inside the coaches as per physician discretion using anatomical triage. The teams addressed, triaged, and identified every injured person and reported to the casualty clearing station officer. The deceased patients all had lesions incompatible with life and were pronounced dead at the scene. Most patients were able to walk from the train to stretchers alongside the train; however, some patients unable to walk by themselves were transported from the train on scoop stretchers carried by Fire & Rescue personnel. The stretchers were carried to the ambulances on temporary bridges positioned between the walkway and the motorway bridge. Additional Fire & Rescue personnel aided in this transportation of the patients.

### Patient treatment and management

Fifteen patients received treatment on the scene or en route to the hospital. The majority had suffered minor bruises, dislocations, and extremity fractures. Because of the weather conditions with gusts of strong gale, it was considered that transport of patients across the East Bridge with a passage height of 65 m was dangerous. Thus, a decision was made that all patients should be admitted to hospitals to the west of the incident. Following discussions with the physician in charge at the EMCC and after acceptance from the head of the emergency department at Odense University Hospital, it was decided that all patients were admitted to Odense University Hospital. As no patients required transport over longer distances and as the character of the injuries in the patients did not require extensive medical treatment at the scene, the HEMS helicopters were cancelled on-scene. Furthermore, a helicopter from the Joint Rescue Coordination Centre was cancelled by the Joint Incident Command before it arrived. Three patients presented themselves with minor injuries to the Emergency Room of Odense University Hospital on their own accord later on the day of the accident.

### Patient evacuation

Due to the structural damage to the easternmost train set, and since power and heating were functional in the westernmost train set, the Joint Incident Command decided to gather the unscathed passengers here to await evacuation. The Local Operational Staff and the Joint Incident Command coordinated transport from the bridge in five tourist busses. The passengers stayed inside the train until the busses arrived to avoid exposure to the wind and the low temperature.

### Evacuation & relatives centre

The Evacuation and Relatives Centre was established by the municipality in Nyborg at request from the Local Operational Staff. The purpose was registration and questioning by police officials and psychosocial support offered by a dedicated team of psychiatrists and psychologists from Odense University Hospital. This psychosocial support team was activated by the physician in the EMCC according to already existing guidelines. The authorities commissioned the Evacuation and Relatives Centre in a sports arena where blankets and food were made available. One MECU and one ambulance were released from the incident site by the Medical Incident Commander to treat minor bruises and assist the police officials. The traffic congestion formed by the closing of the bridge delayed the arrival of the psychosocial support team to the Evacuation and Relatives Centre substantially and unfortunately, the majority of the passengers had left the center before the arrival of the psychosocial support team.

### SINE communication

The major problem was difficulties in hearing the SINE radios due to wind noise. The use of earplugs was not standard for the EMS personnel at the time of the MI. Secondly, according to EMCC and CFB logs, 61.1 percent of the 36 EMS radios were used as intended as per the SINE Major Incidence radio net grid. Nineteen percent of the radios switched to the wrong interdisciplinary talk group 198, primarily the Joint Incident Command channel instead of the HEALTH channel. Another nineteen percent of the radios did not switch as intended, e.g. switched too late or as the result of an intended breach in MI grid adherence, e.g. the Casualty Clearing Station Officer talking to EMCC instead of Medical Incident Commander. The Joint Incident Command all switched as intended and communication within incident managers as for the Casualty Clearing Station personnel was reported unproblematic in the debriefings.

### Prehospital patient medical record system

The prehospital patient medical record system was used as an electronic casualty clearing station record. Patients treated and transported were registered in this record system. For a unique identification of the patients, a wristband with barcodes was optically scanned and entered into the patient record system. When the ambulance departed, the record system kept scores of patients, secured information for the receiving hospital, and gave sufficient documentation of patient treatment.

### Defusing of EMS personnel

The EMS succeeded in defusing three ambulances and one MECU crew after the incident. One of the MECU prehospital physicians conducted the defusing. For the remaining units, lack of time and different priorities prevented defusing.

### Debriefing of EMS personnel, survivors, and relatives

Three weeks after the incident, three meetings with structured debriefing were held. On two occasions, debriefing of the relatives to the deceased patients and the surviving passengers took place. The third meeting was directed towards all the EMS personnel that were involved. Professionals from the EMCC as well as EMS personnel and the psychosocial support teams attended the debriefing. At the meeting, psychologists investigating the psychological effects of MI addressed the EMS personnel afterward for interviews. The results of the study remain to be published.

### Press and media

From Danish media insight platform Infomedia®, a search revealed that newspapers, television, and internet-based media have issued 861 pressed or online articles, notes, and television reports regarding the incident itself and the subsequent investigations [19, 20] in the causes for the trailer detachment from the wagon. Several TV programs have described the events and the aftermath of victims, relatives, and witnesses from the Great Belt Train Accident including a documentary [21] on Danish National Television two years after the incident.

### The aftermath

Based on the investigations by the Accident Investigation Board [19], a report concluded that it was highly probable that the semi-trailer was loaded correctly with the kingpin placed in the saddle, but that the lock that was supposed to secure the semi-trailer to the pocket wagon was not working correctly. Therefore, the semi-trailer did not lock securely to the pocket wagon, and subsequently fell off because of the wind pressure and struck the passing high-speed train, causing a MI.

As a direct result of the debriefings, it was decided to purchase earplugs for all MECUs in the Region of South Denmark to avoid problems with hearing the radio transmissions due to wind noise.

A report by Jørgensen et al. [22] on the psychological repercussions in passengers and relatives from the accident concluded that nineteen percent suffered Post Traumatic Stress Disorder five months after the accident and that twenty-two percent of the participants displayed clinically significant symptoms of depression.

## Discussion

### Summary of events

The Great Belt train accident was handled according to national guidelines by Danish major incident preparedness as the result of a solid major incident management concept and substantial training.

The collision between the high-speed train and the trailer resulted in substantial damage, the killing of eight people while fifteen patients were admitted to a trauma center. The EMS response was prompt but challenged by difficult access to the incident site. EMCC dispatched nineteen EMS units in the MI response. The organization of the incident site was partly improvised and took advantage of the unusual closing of the essential link between the two major parts of Denmark. However, it was still handled according to the framework in the REFIL [14] and probably saved time for transport to a casualty clearing station on land that authorities are training in annual Great Belt exercises [23]. Furthermore, the wind conditions made it impossible to set up tents for the casualty clearing station as stated in the official evaluation [20] of the EMS response.

There were five experienced EMS physicians involved in the incident, all with formal education in MI management. The debriefing reported that patient management was uncomplicated, and because of ample resources and ideal incident site organization, the logistics of the MI were considered well organized.

Surge capacity at the receiving hospital was no factor due to the limited number of patients and because of the declaration of a MI by preparedness leaders at OUH that prompted the activation of the response plan, based on the initial reports from the Medical Incident Commander.

The subsequent debriefing of personnel was carried out in accordance with existing guidelines [14]. The public outreach in terms of the two debriefing sessions with patients and with relatives was optional and probably caused by public demand due to the unique character of the incident. Initial defusing was only partly successful since just four of nineteen EMS units were able to perform the defusing.

### The EMS challenges of the Great Belt Train Accident

#### Access

The time from dispatch to arrival at the scene was compromised for several of the arriving EMS units because of the congestion on the motorway due to the closure of the bridge at both sides of the connection. The queues stretched for almost ten kilometers from the abutment of the bridge. With a daily average of 36.000 cars passing the Great Belt Bridge, congestion is a common problem during closure. There is no alternate connection nearby and once stuck in a queue, it is impossible to leave the motorway.

Fortunately, the closed motorway bridge was available and was used for the organization of the incident site and positioning of MECUs, ambulances, Fire & Rescue, and Police vehicles. The two HEMS helicopters landed on the bridge 50–100 m from the train. Therefore, scene safety was considered optimal and provided a perfect setup in the actual situation with very a short distance from the train to the ambulances which eased the patient management. Furthermore, the generally mild nature of the injuries in the patients made triage and evacuation easy.

#### Weather conditions

The harsh weather conditions with low temperature and heavy winds provided a challenge for the EMS. The MECU and ambulances dispatched from Region Zealand had to cross the East Bridge in wind speeds at up to 20 m/sec in 75 m height. In the debriefings, the EMS personnel expressed concerns and explained that they had to reduce speed and drive in the middle of the motorway. Therefore, the weather conditions may potentially have compromised the safety of the EMS personnel and indeed did influence the transportation of the patients away from the accident site.

The flood warning issued by the Danish meteorological institute had prompted that the Local Operational Staff of the Funen Police District was established and already functional when the accident happened.

The decision to collect physically unharmed passengers in the train set with power and heating and to keep them there until evacuation in buses was possible, relied solely upon the weather conditions and the fact that all passengers had been triaged and managed by EMS triage teams.

#### Communication

Communication breakdown is often seen in MI [5, 33, 34] due to network overload or outdated technology. This was not the case in the Great Belt Train Accident.

However, there seems to be a challenge in using the SINE as intended. A CFB report from 2018 [24] concluded that guideline adherence for the interdisciplinary use of SINE was poor. Based on 450 interviews of police, fire & rescue, EMCC, and EMS personnel, it was evident that knowledge and experience were varying, and especially the EMS personnel and the EMCC personnel used SINE in a restricted manner that did not fully utilize the options and possibilities of the system. A study by Holm [25] among Danish prehospital physicians concluded that initial training in the use of communication devices had not been prioritized as highly as other technical skills, but found the training level to be sufficient for everyday use for the majority of users. For a substantial minority, however, further training was considered necessary.

Therefore, despite a well-designed framework, substantial national investments, and the continuing development of the network, the use of the communication devices is not optimal and in this case potentially problematic. In an even more complex incident, command and control might have been compromised, potentially affecting the MI management and subsequently tampered the survival of patients and the security of EMS personnel. One explanation of the partly inappropriate use of SINE communication could be the interface design of the SINE radios that may be overwhelming and non-intuitive when not used routinely or in a stressful environment or situation.

When faced with a MI, the startle effect may strike EMS personnel, i.e. when faced with sudden, unexpected impressions, an individual may respond with compromised performance ranging from distraction to inappropriate actions or hasty decision-making. Well-learned procedures and skills can be discarded and substituted by inappropriate reactions, including freezing or over-reacting. In the debriefing, EMS personnel described their skepticism when receiving the mission; Is this an exercise? A test? Therefore, to mobilize sufficient mental capacity to operate a complex, non-intuitive radio setup is challenging and may lead to mistakes and inappropriate use of communication systems. On the other end of that spectrum is the EMS professional that is self-assured: “I have trained for this. I have even carried out a full-scale exercise with the exact same scenario. I know what to do.” These opposite or extreme positions may have accounted for the predominant guideline adherence and a minority having difficulties with using communication devices as intended. To improve communication in Danish MI and EMS in general, the CFB report [24] recommends the use of patching or forced steering of radios from EMCC, i.e. an EMCC operator patches all units for the same task in a PC based grid. Patching is standard in Norway and other countries. The reluctance to use patching in EMS communication is most likely to be funding-related. It will require a dedicated 24/7 operator in the five regional EMCCs at an estimated 2 million Euros per year in total.

#### Patient flow

The medical task in the Great Belt MI turned out to be manageable due to the nature of the injuries sustained by the train passengers. The EMCC physician decided to allocate all passengers to the same hospital, once the Medical Incident Commander established the magnitude of the MI. Reports from other European major incidents, [26–29] underline command and control in every phase as the essential component in MI management. The reports all mention the need for continuous communication from the incident site(s) to the receiving hospital(s) and the need for overall steering of patient flow to avoid crowding at specialist hospitals with mildly or moderately injured patients. The electronic casualty clearing station record was an essential tool for all parties to have command and control of patient management.

In this case, the hospital surge capacity was sufficient. Previous studies [30, 31] have described the need for contingency preparedness plans within the hospitals. The transition from pre- to in-hospital phase in MI should be seamless and requires detailed preparedness plans and predefined systems for trauma management, e.g. Advanced Trauma Life Support [32] (ATLS), which is the preferred system in Denmark.

#### Major incident reporting

Most lessons learned from MI stem from safety boards [19], commissions [20, 33], and government hearings [34, 35]. Therefore, delayed or even lost information is inherent when purveying the experiences and potential misfits to improve future MI management [36]. Professionals may discuss their findings and experiences informally, but for necessary MI management changes to occur; there is a need for a more formal platform.

The Major Incident Reporting Collaborators developed a template for reporting the medical pre-hospital response to MI [37]. However, implementation and dissemination of the MI reporting have been challenging due to funding, technical issues, language barriers, and time delay in receiving completed reports. Furthermore, relevant considerations whether society preparedness becomes vulnerable when exposing weaknesses in MI management may hinder MI reporting.

The inherent problem that no formal obligation to report after major incidents exist seems to be essential. Furthermore, complex data protection legislation prohibits the publication of incidents with less than six patients. The authors find it to be “a moral and ethical imperative to disseminate experiences and lessons learned after participating in MI, in the interest of future colleagues and victims”. Some professionals may be reluctant to report MI on open access platforms because of a fear of revealing sensitive information to terrorists. This fear may be exaggerated and the result of a general fear of terrorist attacks [38, 39] seen in Europe in recent years.

#### Formal education in major incident management

There is a bottleneck challenge in terms of providing formal education in MI management to all Danish prehospital physicians. Thirty prehospital units (Twenty-six MECUs and four HEMS helicopters) are on 24/7/365 service in Denmark. In the Danish HEMS, MI certification is mandatory. However, there are only sixteen to twenty seats available at the MI management course each year. Therefore, 48% of Danish prehospital physicians are not certified in MI management (Table 1). In comparison, all police and Fire & Rescue incident commanders are certified.

Despite a formal education, the rare occurrence of MI represents a challenge in terms of lack of routine. A paper by Johnsen et al. [40] reported MI in just 0.16% of HEMS/SAR missions. Due to the medical and logistical capabilities of rotor-wing aircraft, the study recommends the use of HEMS/SAR capabilities in MI, which is standard in Denmark and thus was available in this Great Belt train accident.

#### Strengths and limitations of the study

The strength of this paper is data consistency and availability of all relevant data from encrypted platforms, providing high data quality. The comprehensiveness of the sources and databases used provides a nearly complete picture of the efforts of the EMS response. This is to our knowledge the first study that describes EMS communication in a MI in detail with affiliation times and actual radio switches.

There are several limitations to the study, including selection bias inherent in observational studies, preventing the determination of a causal relationship between the EMS response and outcome. Information bias and confounding may also affect the interpretation of the results.

The Danish authors of this study all played major roles as Medical Incident Commander, Casualty Clearing Station Officer, and Health sector representative in the Local Operational Staff. This may have produced recall bias but also contributed to the completeness of the operational descriptions.

The generalisability of this study may be substantial for other pre-hospital critical care organizations operating in comparable geopolitical settings.

## Conclusion

The Great Belt train accident on January 2^nd^, 2019 prompted a massive prehospital response that involved nineteen EMS units and the declaration of a major incident. Multiple national authorities were activated and the Danish major incident preparedness was tested. Fortunately, the system proved to function well and the major incident management was successful despite severe weather conditions in a hostile environment on a bridge. The overall EMS performance was considered according to national guidelines and was the result of robust preparedness, a comprehensive major incident management concept, and finally substantial training.

There was a delay in time to triage and treatment because of safety issues and difficult access to the train. Interdisciplinary communication was somewhat compromised despite a robust radio system. This is to some extent concerning, since the potential challenge was previously described. Interdisciplinary radio communication remains an area for potential improvement. There is a need for an expansion of capacity in formal education in MI management in Denmark.

## Supplementary Information

**Additional file 1**. Overview of EMS units tasked for incident. AMB: Ambulance; MECU: Mobile Emergency Care Unit; HELI: Helicopter; STD DEV: Standard Deviation.

**Additional file 2**. Prehospital Patient Journal (PPJ). Electronic Casualty Clearing Station and patient wristband. PPJ: Prehospital patient journal.

**Additional file 3**. Aerial photo of incident site. Private photo.

**Additional file 4**. Aerial photo of incident site. Private photo.

## Data Availability

An anonymized dataset with relevant variables supporting the findings is available from the corresponding author on reasonable request.

## Abbreviations

MI: Major incident
EMS: Emergency medical services
EMCC: Emergency medical communication center
EMT: Emergency medical technician
MECU: Mobile emergency care unit
HEMS: Helicopter emergency medical service
SAR: Search and rescue
DEMA: Danish Emergency Management Agency
DHA: Danish Health Authority
CFB: Centre of Emergency Communication
