# Supplementary material for: The Great Belt train accident: the emergency medical services response
Source: Scand J Trauma Resusc Emerg Med. 2021 Sep 23;29:140. doi: 10.1186/s13049-021-00954-7 (PMC8461896; doi:10.1186/s13049-021-00954-7)
Supplement: Supplementary file 1 — Additional file 1. Overview of EMS units tasked for incident. AMB: Ambulance; MECU: Mobile Emergency Care Unit; HELI: Helicopter; STD DEV: Standard Deviation. [file 13049_2021_954_MOESM1_ESM.docx]

Additional material 1.

Overview of EMS units tasked for incident.

AMB: Ambulance; MECU: Mobile Emergency Care Unit; HELI: Helicopter; STD DEV: Standard Deviation
