# Supplementary material for: The Great Belt train accident: the emergency medical services response
Source: Scand J Trauma Resusc Emerg Med. 2021 Sep 23;29:140. doi: 10.1186/s13049-021-00954-7 (PMC8461896; doi:10.1186/s13049-021-00954-7)
Supplement: Supplementary file 2 — Additional file 2. Prehospital Patient Journal (PPJ). Electronic Casualty Clearing Station and patient wristband. PPJ: Prehospital patient journal. [file 13049_2021_954_MOESM2_ESM.docx]

Additional material 2.

Prehospital Patient Journal (PPJ). Electronic Casualty Clearing Station and patient wristband.

**
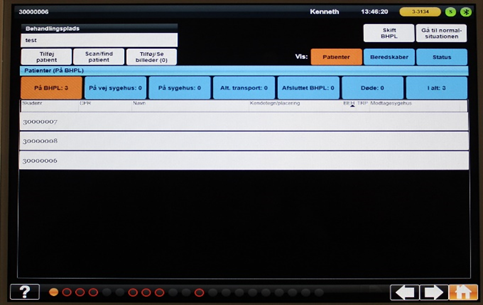

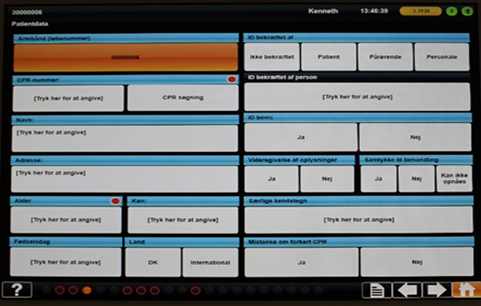
**

**
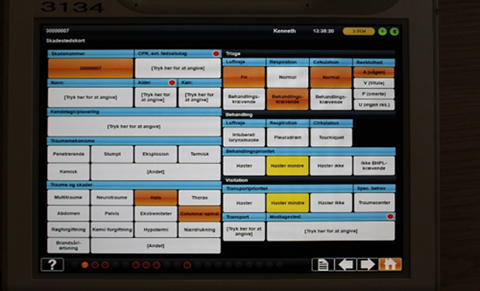

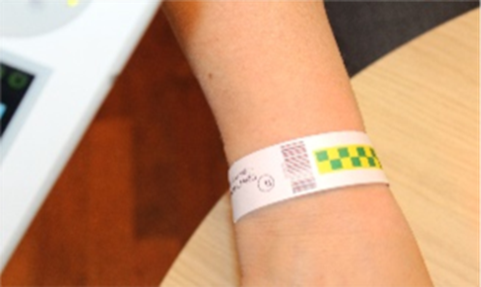
**

PPJ: Prehospital Patient Journal.
