# Supplementary figures and images for: The Great Belt train accident: the emergency medical services response
Source: Scand J Trauma Resusc Emerg Med. 2021 Sep 23;29:140. doi: 10.1186/s13049-021-00954-7 (PMC8461896; doi:10.1186/s13049-021-00954-7)

Additional material 3.

Aerial photo of incident site. Private photo.


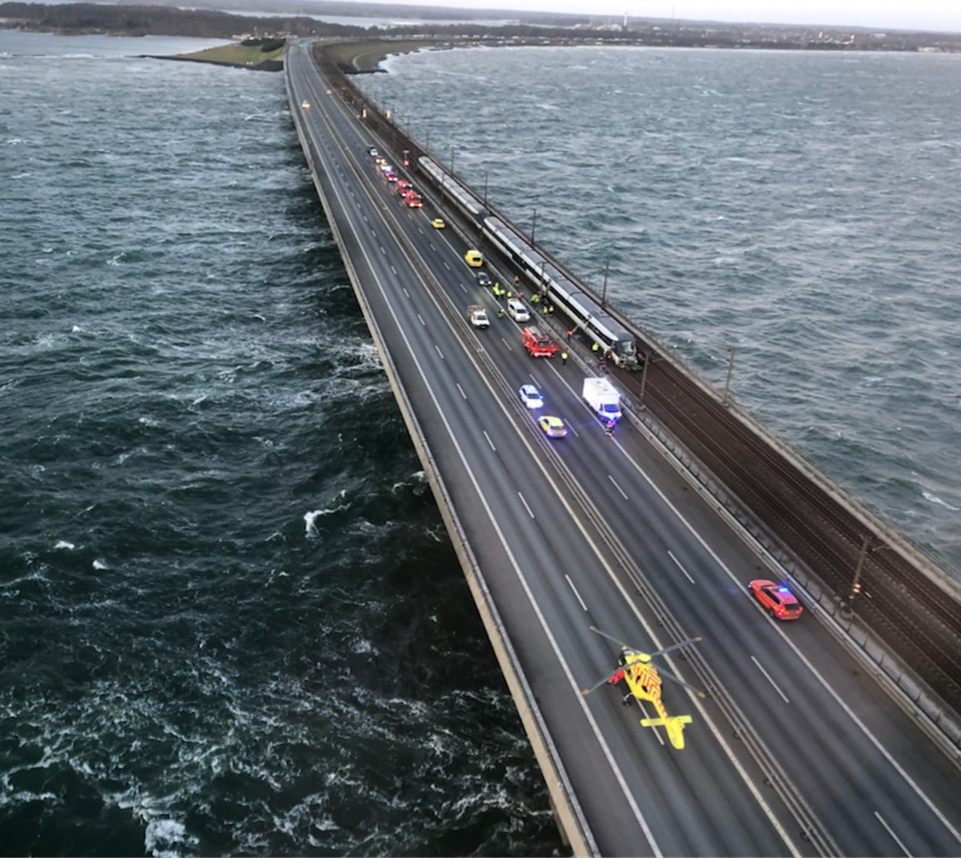

Supplement: Supplementary file 3 — Additional file 3. Aerial photo of incident site. Private photo. [file 13049_2021_954_MOESM3_ESM.docx]

Additional material 4.

Aerial photo of incident site. Private photo.


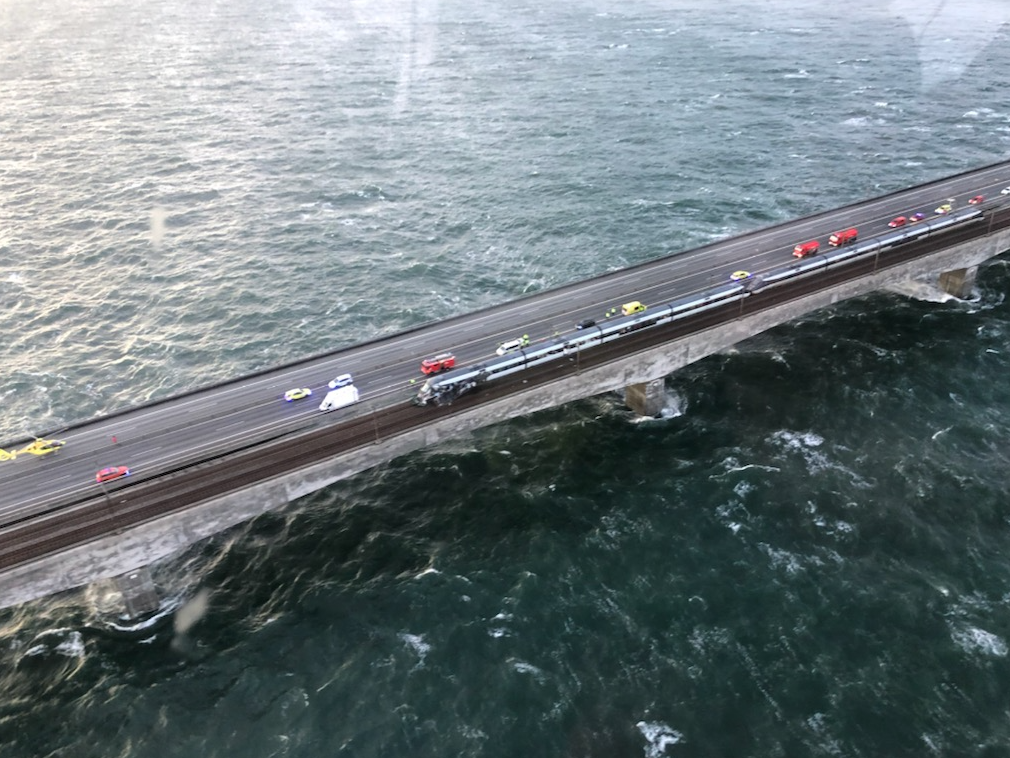

Supplement: Supplementary file 4 — Additional file 4. Aerial photo of incident site. Private photo. [file 13049_2021_954_MOESM4_ESM.docx]
